# Vignette-Based Reflections to Inform Genetic Testing Policies in Living Kidney Donors

**DOI:** 10.3390/genes13040592

**Published:** 2022-03-26

**Authors:** Gurmukteshwar Singh, Reginald Gohh, Dinah Clark, Kartik Kalra, Manoj Das, Gitana Bradauskaite, Anthony J. Bleyer, Bekir Tanriover, Alex R. Chang, Prince M. Anand

**Affiliations:** 1 Department of Nephrology, Geisinger Health, Danville, PA 17822, USA; kkalra@geisinger.edu (K.K.); mdas1@geisinger.edu (M.D.); achang@geisinger.edu (A.R.C.); 2 Division of Organ Transplantation, Rhode Island Hospital, Providence, RI 02908, USA; reginald.gohh@brownphysicians.org; 3 Natera, Inc., Austin, TX 78753, USA; dclark@natera.com; 4 Division of Nephrology, Einstein Medical Center, Philadelphia, PA 19141, USA; bradausg@einstein.edu; 5 Division of Nephrology, Wake Forest School of Medicine, Winston-Salem, NC 27157, USA; ableyer@wakehealth.edu; 6 Division of Nephrology, University of Arizona College of Medicine, Tucson, AZ 85719, USA; btanriover@arizona.edu; 7 Department of Nephrology, Medical University of South Carolina, Charleston, SC 29425, USA; mohanp@musc.edu

**Keywords:** genetic testing, living kidney donor, kidney donation, family history of kidney disease

## Abstract

Family history of kidney disease increases risk of end-stage kidney disease (ESKD) in donors. Pre-donation genetic testing is recommended in evaluation guidelines and regulatory policy. Collaborating across several institutions, we describe cases to illustrate the utility as well as practical issues in incorporating genetic testing in transplant protocols. Case 1 is from 2009, before pervasive genetic testing. A healthy 27-year-old Caucasian male had an uneventful donor evaluation for his mother, who had early onset ESKD of unclear cause. He participated in paired-exchange kidney donation, but developed progressive kidney disease and gout over the next 10 years. A uromodulin gene mutation (NM_003361.3(*UMOD*):c.377 G>A p.C126Y) was detected and kidney biopsy showed tubulointerstitial kidney disease. The patient subsequently required kidney transplantation himself. Case 2 was a 36-year-old African American female who had an uneventful kidney donor evaluation. She underwent gene panel-based testing to rule out ApolipoproteinL1 risk variants, for which was negative. Incidentally, a sickle-cell trait (NM_000518.5(*HBB*):c.20A>T p.Glu7Val) was noted, and she was declined for kidney donation. This led to significant patient anguish. Case 3 was a 26-year-old Caucasian female who underwent panel-based testing because the potential recipient, her cousin, carried a variant of uncertain significance in the hepatocyte nuclear factor-1-β (*HNF1B*) gene. While the potential donor did not harbor this variant, she was found to have a likely pathogenic variant in complement factor I (NM_000204.4(*CFI*):c.1311dup:p.Asp438Argfs*8), precluding kidney donation. Our cases emphasize that while genetic testing can be invaluable in donor evaluation, transplant centers should utilize detailed informed consent, develop care pathways for secondary genetic findings, and share experience to develop best practices around genetic testing in donors.

## 1. Introduction

While overall, kidney donor outcomes are excellent, analyses of Norwegian and American cohorts show a more than 10-fold increase in subsequent development of end-stage kidney disease (ESKD) in donors compared to nondonors [1,2]. Among more than 100,000 living kidney donors, ESKD risk was almost 2-fold greater if they had a first-degree biological relationship to the recipient [3]. Closer donor–recipient biological relationships, such as identical twins, have a higher risk compared to parents and other siblings [4]. Moreover, the donor–recipient biological relationship is associated with a higher risk of allograft failure in Black recipients, further underscoring the role of inherited kidney disease in transplant outcomes [5].

The yield and accessibility of genetic testing for inherited kidney diseases are rapidly evolving. In a cohort of more than 3000 kidney disease patients, exome sequencing led to a genetic diagnosis in nearly 10% [6]. More than 500 monogenic causes of chronic kidney disease (CKD) have been identified, making genetic testing an important precision-medicine tool [7]. Early and appropriate use of genetic testing has a high diagnostic yield and can save thousands of dollars in healthcare costs [8]. Moreover, the cost of human-genome sequencing has been declining dramatically, recently dropping below $1000 [9].

Recognizing the risk for inherited kidney disease in donors, Kidney Disease Improving Global Outcomes (KDIGO) guidelines recommend appropriate genetic history and testing during kidney donor evaluation [10]. The Organ Procurement and Transplantation Network (OPTN) policies concur, but acknowledge discovery of previously unknown genetic findings as a secondary risk [11]. In 2020, KDIGO clinical practice guidelines on the evaluation and management of potential kidney recipients recommended utilizing pre-transplant genetic testing according to etiology of ESKD. For instance, targeted genetic testing is recommended for focal segmental glomerulosclerosis, C3 glomerulopathy and atypical hemolytic uremic syndrome [12]. However, detailed guidelines and best practices for genetic testing have not been clearly defined in biological relatives wishing to donate a kidney to recipients with these conditions.

Some transplant centers have attempted to bridge this void by developing their own targeted gene panels after literature review [13]. However, these efforts currently remain limited to single-center pilot studies. Expert authors have attempted to curate inherited kidney diseases with clearly identified causal genes and suggested testing in living donor candidates [14]. However, a standardized approach to genetic testing in potential living donors remains elusive. Developing transplant program policies around these issues remains a challenge, with little to no multi-center validation of suggested approaches. We formed a multi-center collaboration to discuss particular areas of uncertainty and reviewed representative patient cases from various centers. Our goal was to identify practical situations that pose significant clinical challenges across centers. Using a vignette-based format, we illustrate three commonly encountered situations that may inform genetic testing for living kidney donors.

## 2. Materials and Methods

We developed a collaboration of general nephrologists, transplant nephrologists and a certified genetic counselor across multiple institutions who share a common interest in genetic risk evaluation in potential living kidney donors. Participants in the collaboration were asked to suggest clinical scenarios from their practice that illustrate the utility and challenges of pre-donation genetic testing and that could inform institutional policy development. The participants identified the following situations as particularly challenging from this standpoint:

- Targeting genetic testing in biological relatives of recipients;
- Variant analysis and determination of pathogenicity;
- Pre- and post-testing genetic counseling;
- Interpretation of incidental or secondary findings on genetic testing;
- Determining significance of genetic results in asymptomatic potential donors.

After identifying these themes, cases best representing them were discussed and three were chosen as the best representative scenarios by consensus. The patients chosen were approached by their clinicians and provided written permission to share their information for the purpose of publication.

## 3. Result

### 3.1. Case Series

#### 3.1.1. Case 1

In 2009–2010, a 27-year-old male Caucasian was evaluated for kidney donation to his mother. He was physically active and had no known chronic conditions. Being incompatible as a donor to his mother due to a high titer of donor-specific antibodies, he agreed to participate in the paired kidney exchange program. He had no proteinuria or hematuria. Baseline serum creatinine was 1.0 mg/dL. A 24 h urine collection showed creatinine clearance of 126 mL/min. He was accepted for paired exchange kidney donation and underwent laparoscopic left donor nephrectomy in 2011.

The donor’s mother had a history of hypertension and gout. She had been diagnosed with stage 3 CKD without proteinuria or hematuria at 39 years of age. Her serologic work-up was negative and serum protein electrophoresis was normal. Kidney ultrasound showed normal-sized kidneys. A cousin had early-onset ESKD from anti-glomerular-basement-membrane disease. A shared decision was made not to pursue a kidney biopsy as it was felt to be of low yield given a normal urine sediment and absent proteinuria. Her CKD progressed and she developed ESKD at 48 years of age. She was listed for kidney transplantation in 2009.

**Figure 1:**
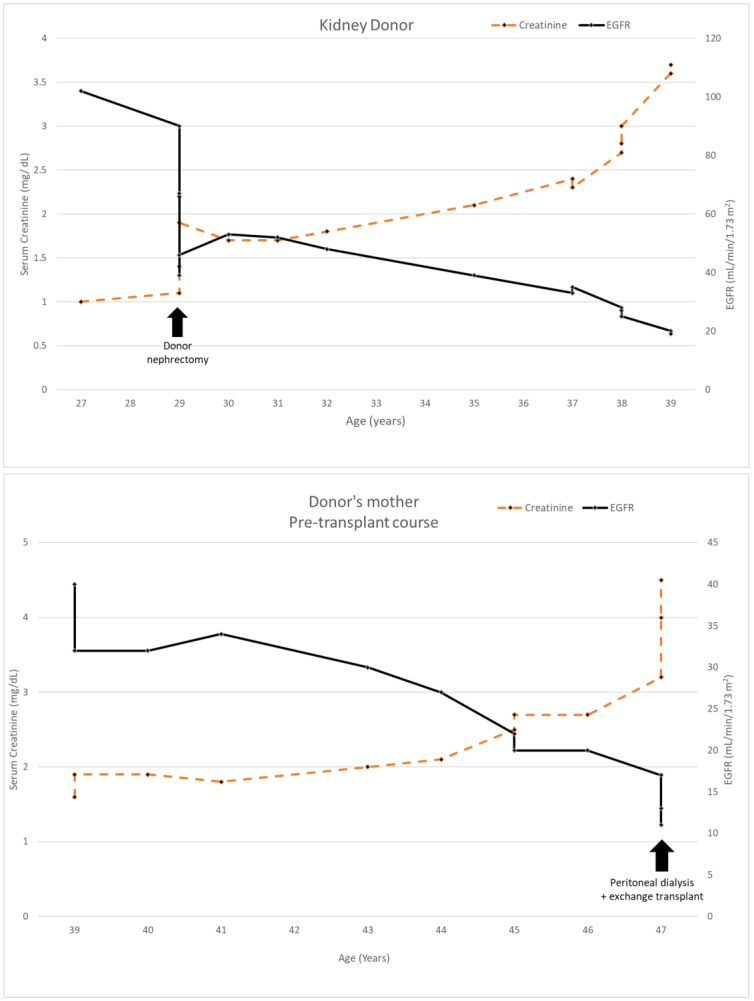
Serum creatinine and EGFR of kidney donor 1 compared to his mother’s clinical course.

**Figure 2:**
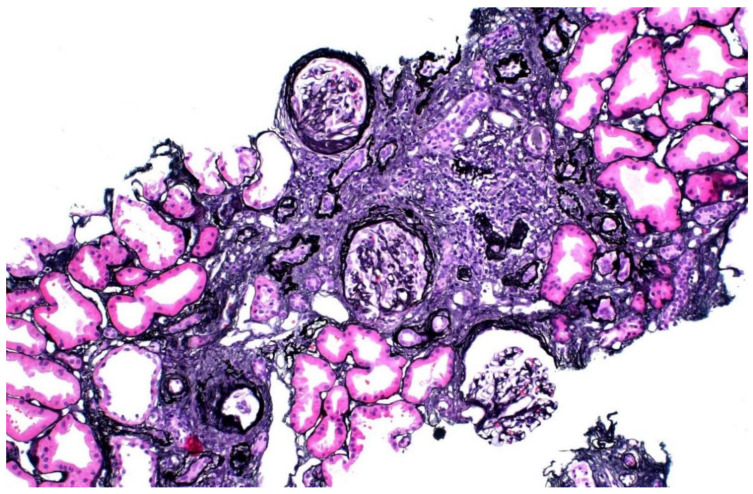
Kidney biopsy of donor 1, showing focal global glomerulosclerosis, arteriosclerosis, tubular atrophy and interstitial fibrosis.

The donor’s serum creatinine stabilized at 1.7–1.8 mg/dL post-nephrectomy. Seen twice in the nephrology clinic during 2013, he subsequently stopped following up. Six years later, he was referred back because his serum creatinine was rising slowly and was up to 2.4 mg/dL. His younger sister had also developed CKD stage 4. He had developed gout and was started on allopurinol. The renal function and estimated glomerular filtration rate (eGFR) of the donor and his mother are shown in Figure 1. A kidney biopsy was performed, and he was referred for genetic testing. His kidney biopsy (Figure 2) showed focal global glomerulosclerosis, arteriosclerosis, tubular atrophy and interstitial fibrosis. Tubular basement membrane showed rupture with interstitial Tamm–Horsfall protein extravasation and associated mononuclear cell infiltration. Whole-exome sequencing showed a likely pathogenic missense variant in the *UMOD* gene (NM_003361.3:c.377 G>A p.C126Y) associated with autosomal dominant tubulointerstitial disease. The same genetic variant was also identified in his mother and sister. He was wait-listed for deceased donor kidney transplantation, and subsequently received a kidney transplant 10 years after having donated one of his kidneys.

#### 3.1.2. Case 2

A 36-year-old African American female underwent evaluation as a living donor for her fiancé. Her medical history was notable for obesity, which had improved following sleeve gastrectomy. She was normotensive and evaluation was otherwise unrevealing. Repeated 24-hour urine creatinine clearances ranged between 119 and 154 mL/min. There were no urinary abnormalities or family history of kidney disease. She was aware of increased kidney disease risk in persons with African ancestry; much of this being attributable to risk alleles in the apolipoprotein L1 gene (*APOL1*) [15]. She agreed to genetic testing to evaluate risk. Testing was performed through the Renasight™ test, a 382-gene panel associated with kidney disease.

Although the patient did not have *APOL1* risk variants, genetic testing identified that she had a sickle-cell trait (NM_000518.5(*HBB*):c.20A>T p.Glu7Val). This result was confirmed by hemoglobin electrophoresis. Due to the increased risk of ESKD associated with this incidental finding, she was turned down for kidney donation. Upon learning this, the patient became upset, stating that she was only tested due to concerns for *APOL1* risk variants. She was concerned that this finding was now part of her permanent medical record. At her request, other regional transplant centers were queried as to whether they would consider her as a donor candidate; all declined to evaluate her. Ultimately, the patient agreed that it was in her best interest not to proceed with kidney donation.

#### 3.1.3. Case 3

A 26-year-old Caucasian female with no history of kidney disease underwent Renasight^TM^ genetic testing as she was considering living kidney donation to her 25-year-old cousin who had a history of CKD of unknown cause and chronic pancreatitis. Her cousin had a heterozygous *HNF1B* variant of uncertain significance (VUS) (NM_000458.4:c.908G>A:p.Arg303His). This variant was also present in the potential donor’s mother and sister, who also had evidence of CKD. Due to concerns that this VUS might be causative, testing was performed in interested living–related kidney donors and as part of family testing to evaluate for co-segregation of the variant with disease.

While the potential donor did not carry the *HNF1B* VUS in question, she was found to be heterozygous for a likely pathogenic variant in complement factor I (NM_000204.4(*CFI*):c.1311dup:p.Asp438Argfs*8). This variant is predicted to cause a frameshift in exon 11 of the *CFI* gene, leading to an out-of-frame transcript and introduction of a premature stop codon. While this truncating variant has not been reported in the literature, similar truncating variants have been reported to be pathogenic [16]. Though this patient had no evidence of atypical hemolytic uremic syndrome (aHUS), she was counseled that having a likely pathogenic variant in *CFI* predisposes individuals to the risk of developing aHUS, although other conditions that trigger complement activation may be needed to cause an acute event. A shared decision was made that it would be safer to avoid living kidney donation due to the potential, uncertain risk that the variant posed.

## 4. Discussion

Our first vignette demonstrates the perils of kidney transplantation in the pre-genetic testing era. It presents a clear argument in favor of aggressive screening for inherited kidney disease in potential kidney donors with a family history of CKD. Meanwhile, our second vignette indicates that such testing may sometimes yield incidental, or secondary findings of clinical significance. The third vignette raises the scenario of finding a likely pathogenic variant when the donor is not exhibiting any clinical features. A summary of the three cases and key challenges/learning points are shown in Table 1. While OPTN living-donor evaluation policies require hospitals to develop and comply with protocols to evaluate for inherited kidney diseases, little guidance is available regarding how to formulate such a protocol and apply it to clinical scenarios [11].

The clinical information yielded by genetic testing in these cases was highly pertinent. Mutations in the *UMOD* gene are the most common cause of autosomal dominant tubulointerstitial kidney disease, resulting in progressive CKD, early onset hyperuricemia and gout [17]. Had genetic testing been readily available in 2009, meticulous testing on donor 1 and his mother would have ruled him out as a kidney donor. For potential living donors with African ancestry, many transplant programs consider *APOL1* testing as the standard of care [15]. Although donor 2 tested negative for these variants, a sickle-cell trait was confirmed as a secondary finding, which led to her being declined as a donor. While kidney dysfunction is commonly associated with sickle-cell disease, there is increasing recognition of kidney vascular disruption, albuminuria, and CKD in patients with sickle-cell trait [18,19]. Among a cohort of 9909 Black patients, sickle-cell trait carriers were at two times the risk of ESKD of noncarriers [20]. Finally, while neither donor 3 nor any of her family members manifested any clinical findings of aHUS, possessing rare coding variants in the *CFI* gene has been associated with reduced complement factor I expression and may lead to impaired complement regulation and increased susceptibility to aHUS [16].

Given the increased kidney risk in donors biologically related to recipients, ordering genetic tests in transplant centers is likely to become more common [3,5,10]. KDIGO recommends genetic testing in donors with a family history suggestive of autosomal dominant inheritance of kidney disease [10]. Based on the first vignette, we recommend re-emphasizing the role of obtaining a family history relevant to kidney disease and pursuing genetic testing in investigating CKD of unknown cause by nephrologists as well as transplant teams. Potential living kidney donors should undergo genetic testing if they have significant family history (e.g., CKD of unknown etiology, cystic kidney disease, congenital disease with extrarenal signs, or aHUS) and receive appropriate genetic counseling. Providing genetic counseling has a vital role in pre-test informed consent, result interpretation and guiding management if pathogenic variants are detected. Even in the absence of a causative variant, a patient should be counseled that a genetic form of disease cannot be ruled out, and, given an informative family history, inferred recurrence risks can be discussed [21].

Our second and third vignettes illustrate the potential for secondary genetic findings. The American College of Medical Genetics working group on secondary findings recommends reporting only known pathogenic variants with a high likelihood of causing disease [22]. Expanded genetic panels have a higher potential for identifying incidental findings that are not directly related to the indication for testing [23]. However, determining the pathogenicity of variants can be difficult for non-geneticists. In a survey, 62% of physicians reported receiving no formal education in genomic medicine, and only 23% felt comfortable discussing genetic risk factors for disease [24]. While well-curated variant lists such as ClinGen may help, knowledge about genomic tests, variant-level analyses, phenotypic-level comparison, and determination of actionability remains critical. Transplant programs should strive for close collaboration with molecular pathologists and geneticists [25].

Pre-genetic testing consent is recommended for potential donors; this covers the potential for receiving incidental or secondary genetic diagnoses, as well as difficulties in obtaining certain kinds of insurance coverage [10]. Most laboratories report having an informed consent process before genomic sequencing. However, there is marked discordance in variant analysis and reporting of secondary findings, complicated by regulations requiring result transparency [26]. Implementing genetic results into nephrology care can be challenging, with additional considerations about insurability, psychosocial implications and availability of genetic counseling [25]. Ultimately, these tasks are the ordering physician’s responsibility, and this responsibility is likely to increase with the use of kidney disease gene panels rather than targeted testing [27]. Finally, to better counsel potential donors, longitudinal studies are needed to better quantify the future kidney risks and other health risks of pathogenic variants that may be identified in currently asymptomatic individuals.

Unfortunately, there remains a paucity of data about how transplant programs are fulfilling these tasks. The most comprehensive experience included 14 potential kidney donors: four were positive for a familial genetic variant, five were negative, and a genetic variant could not be identified in five. Potential recipients were tested to identify a familial variant first, followed by testing of donors [28]. While this is a prudent approach, our cases illustrate that utilizing broad-based gene panels for donor testing can identify significant incidental findings that would otherwise be missed (e.g., sickle-cell trait in our second donor or *CFI* variant in our third donor). Most transplant programs currently choose between these approaches depending on resources and availability. There is a dire need for further studies to describe best-use scenarios of variant-based versus gene panel-based testing in the context of pre-donation evaluation. Moreover, determining the pathogenicity of detected genetic variants remains a demanding process, with imperfect consensus about best practices, making protocol formulation and implementation a challenge [25].

## 5. Conclusions

In conclusion, genetic testing is critical in potential kidney donors with a family history of kidney disease. Institutions should develop an informed consent process and policies to address secondary findings. There is an unmet need to share experience with approaches to genetic testing and best practices for variant analysis across transplant programs.

## Figures and Tables

**Table 1:** The three illustrative cases and key challenges/learning points identified.

Case	Learning Points	Challenges
Uromodulin-related ADTKD resulting in ESKD post-donation	Donor–recipient relationship is an absolute indication for pre-donation genetic testing	Genetic panel-based testing or variant-directed testing
Incidental sickle-cell trait detected on pre-donation testing	Pre- and post-testing genetic counseling is vital	How to determine variant pathogenicity and post-donation implications
Secondary pathogenic finding in asymptomatic potential donor	Genetic panel-based testing may reveal unrelated secondary findings	Management and monitoring of asymptomatic pathogenic variant carrier
ADTKD: Autosomal dominant tubulointerstitial kidney disease; ESKD: End Stage Kidney Disease		

## Data Availability

Not applicable.
